# Detection and phylogenetic analysis of porcine epidemic diarrhea virus in central China based on the ORF3 gene and the S1 gene

**DOI:** 10.1186/s12985-016-0646-8

**Published:** 2016-11-25

**Authors:** Yunfang Su, Yunchao Liu, Yumei Chen, Baolei Zhao, Pengchao Ji, Guangxu Xing, Dawei Jiang, Chang Liu, Yapeng Song, Guoqiang Wang, Dongliang Li, Ruiguang Deng, Gaiping Zhang

**Affiliations:** 1 College of Veterinary Medicine, Northwest Agriculture and Forestry University, Yangling, Shaanxi 712100 China; 2 Henan Provincial Key Laboratory of Animal Immunology, Henan Academy of Agricultural Science, Zhengzhou, 450002 China; 3 College of Animal Science and Veterinary Medicine, Henan Agricultural University, Zhengzhou, 450002 China; 4 Jiangsu Co-innovation Center for Prevention and Control of Important Animal Infectious Diseases and Zoonoses, Yangzhou, 225009 China

**Keywords:** PEDV, ORF3 gene, S1 gene, Phylogenetic analysis, Variants

## Abstract

**Background:**

Porcine epidemic diarrhea (PED) has increased in severity in China since 2010. To investigate further the infectivity, genetic diversity and molecular epidemiology of its causative agent, the porcine epidemic diarrhea virus (PEDV), we assessed 129 clinical samples, which were the intestinal tissue of piglets with severe diarrhea, from 17 cities in central China. Both the spike (S) glycoprotein (S1, 1–789 amino acids (aa)) and the full-length ORF3 gene of 21 representative field strains from 21 farms in 11 cities were sequenced and analysed.

**Methods:**

PEDV was detected by reverse transcription-polymerase chain reaction (RT-PCR), and S1 and ORF3 sequences were processed by the Clustal W method via DNAMAN 8 software, and phylogenetic trees were constructed by the neighbor-joining method using MEGA 6 software.

**Results:**

The prevalence of PEDV was 92.25% and was detected in 119 of 129 samples, with 94.03% (63 of 67) of pig farms harbouring the disease. According to the phylogenetic analysis of the S1 genes, our isolates all fell into group G2 (variants) and showed a close relationship to isolates from Chinese (HN1303, CH/ZMDZY/11 and AJ1102), Korean (AD01), American (MN, IA1, IA2 and 13–019349) sources, and these isolates differed genetically from other Chinese (LZC, CH/HNZZ/2011 and SD-M) and Korean (SM98) strains as well Japanese (83-P5 and MK) strains. In addition, our isolates differed from attenuated vaccine strains, CV777 (used in China) and DR13 (used in Korea). According to our derived amino acid sequence analysis, we detected one novel variant PEDV, viz: CH/HNLY, with 4-aa insertion/deletion (RSSS/T) at position 375 and 1-aa (D) deletion at position 430 compared to the CV777 attenuated strain. These mutations were located on the receptor binding domain. Our ORF3 gene analyses showed that the prevalent PEDV isolates were variants, and the isolated strains differed genetically from the vaccine strains.

**Conclusions:**

These findings illustrated the existence of genetic diversity among geographically distinct PEDV strains, and our study has provided an impetus to conduct further research on the PEDV receptor binding protein and on the new and efficacious vaccines design.

**Electronic supplementary material:**

The online version of this article (doi:10.1186/s12985-016-0646-8) contains supplementary material, which is available to authorized users.

## Background

Porcine epidemic diarrhoea (PED) is an acute, highly contagious disease of swine caused by the PED virus (PEDV), which leads to severe vomiting and diarrhea along with dehydration and high mortality in new-born piglets [1]. PEDV belongs to the family *Coronaviridae*, genus *Alphacoronavirus* and was first reported in England [2]. PEDV has now been reported worldwide, including in Belgium, France, Japan, Korea, Italy, Thailand, USA, Canada and Mexico [1–5] and poses severe economic burdens. Since 2010 serious PED has been detected in China [6]. PEDV is an enveloped ssRNA coronavirus with a 28 kb genome, including seven open reading frames (ORFs), a 5′ untranslated region (UTR), and a 3′ UTR with a polyadenylated tail. The seven ORFs encode four structural proteins, spike (S), envelope (E), membrane (M) and nucleocapsid (N) and three non-structural proteins, replicases 1a, 1b and ORF3 [7, 8]. The S protein contains a specific receptor binding site that is important for cell membrane fusion and virus entry and is an antigenic target for neutralising antibodies [9]. The M protein is the most abundant surface protein, and coexpression with E protein to form pseudo-particles results in interfering genic activity [10]. The N protein is highly conserved and binds to virion RNA to provide a structural basis for the helical nucleocapsid, and it is used for early diagnosis [11]. For non-structural proteins, replicases 1a and 1b are multi-functional and associated with viral genome replication [12], and the accessory ORF3 protein is thought to influence virulence [13].

The S1 domain (amino acids (aa) 21–793) contains two subdomains: NTD (aa 21–324) and CTD (aa 253–638) [14], with the latter binding to porcine aminopeptidase N (pAPN) which is important for cell membrane fusion and virus entry and it is the antigenic target of neutralising antibodies [15]. Thus, the variable S1 gene has been widely used for studies of PEDV genetic evolution and diversity [16, 17]. PEDV CV777 has three main neutralising epitopes: aa 498–637 (CO-26 K equivalent, COE gene) [18], aa 747–754 (YSNIGVCK) and aa 763–770 (LQDGQVKI) [19]. Sequence analyses indicated that the latest PEDV isolated strains in China were different from attenuated CV777 [16, 20, 21]. Multiple mutations of the S protein resulted in two PEDV genotypes, G1 and G2 (classical strains and variant strains) [16], and a recent study suggested that antigenic variation exists between G1 and G2 [22]. The variable S1 gene has been widely used for studies of PEDV genetic evolution and diversity [16, 17]. The accessory ORF3 gene is highly relevant to the virulence of PEDV as previously stated [13], and reduction in virulence is produced through cell culture adaptation [23, 24]. The ORF3 gene of attenuated vaccine isolates has a continuous deletion of 17 amino acids (aa 82–99), thus distinguishing the vaccine isolates from variant PEDV [24]. Therefore, the ORF3 gene has been the focus of molecular epidemiology PEDV studies [16, 20, 21, 25]. As aforementioned, we chose the S1 and ORF3 genes as the target genes for phylogenetic analysis.

Phylogenetic analysis of variations in the S1 gene of isolates collected in China distinguished the PEDV genotypes G1 and G2, including 1a classical PEDV, 1b classical PEDV, 2a circulating PEDV and 2b circulating PEDV subgroups [26]. Based on the ORF3 gene, the phylogenetic analysis of PEDV isolates in China manifested in genotypes G1 and G2, the G2 genotype including 2a PEDV and 2b PEDV subgroups [27]. To investigate further infectivity, genetic diversity and molecular epidemiology of PEDV, we performed phylogenetic analyses based on the S1 and ORF3 genes of the latest Chinese isolates collected. In this study, 129 samples were obtained from 67 farms in 17 cities of central China, and the S1 and ORF3 genes of 21 representative field strains from 21 farms in 11 cities were sequenced and analysed.

## Methods

### Sample collection and cDNA synthesis

In this study, 129 intestinal tissue samples from new-born piglets suffering from severe diarrhea were collected from 67 farms in 17 cities (Zhengzhou, Kaifeng, Anyang, Hebi, Puyang, Xinxiang, Luoyang, Nanyang, Pingdingshan, Sanmenxia, Luohe, Jiaozuo, Xuchang, Yuncheng, Zhoukou, Zhumadia, and Xinyang) of central China from July 2014 to July 2015. Samples were diluted with 5 volumes of 0.9% saline (w/v), frozen and thawed three times and then clarified by centrifugation for 5 min at 3000 rpm. Three hundred microliters of the supernatants were used for RNA extraction using TRIzol, dissolved in RNase-free water and then stored at −80 °C until further use. Synthesis of the cDNA was carried out through reverse transcription as described below. A total of 13 μl of viral RNA (approximately 1 μg) was mixed with 1 μl of 10 pmol Oligo (dT) primer (TaKaRa), incubated at 70 °C for 10 min, then placed on ice for 1 min. Next, 4 μl of 5× RT buffer, 1 μl of dNTP (2.5 mM) mixture, 0.5 μl of RNase inhibitor (40 U/μl) and 0.5 μl of reverse transcriptase M-MLV (200 U/μl) were added and gently mixed. The mixture was kept at 42 °C for 1 h and the resulting cDNA stored at −20 °C until further use.

### Clinical samples detection

Primers used in this study were designed to target the conserved regions of the S gene and were synthesised by Sangon Biotech. The primers are listed in Table 1. For the PCR reactions, 1 μl of cDNA, 10 μl ExTaq DNA polymerase (TAKARA), 1 μl of each primer (10 pmol) and RNase-free water in a total volume of 20 μl. The amplification was carried out as follows: 95 °C for 5 min, followed by 33 cycles of 95 °C for 1 min, 57 °C for 1 min and 72 °C for 1 min and finally 72 °C for 10 min. The products were examined by electrophoresis using a 1.0% agarose gel.

**Table 1:** Primers used in this study

Primer name	Nucleotide sequence, 5′-3′	Size(bp)	Primer location^a^
PEDV-F	TTTATTCTGTCACGCCAT	197	2,2709–22,726
PEDV-R	AGATTTACAAACACCTATGTTA		22,884–22,905
S1U1-F	GGTAAGTTGCTAGTGCGTAA	1461	20,570–20,589
S1U1-R	CAGGATCATCACAATAAAGAA		22,010–22,030
S1U2-F	TTTCTGGACCATAGCATC	1117	21,939–21,956
S1U2-R	AGCACAATCAACACTAAC		23,038–23,055
ORF3-F	TCCTAGACTTCAACCTTACG	833	24,741–24,760
ORF3-R	GGTGACAAGTGAAGCACAGA		25,551–25,570

^a^In relation to the genome of PEDV CV777 strain (AF353511)

### Sequencing of the S1 and ORF3 genes

The S1 and ORF3 genes of 21 representative field strains from 21 farms in 11 cities (Kaifeng, Anyang, Hebi, Puyang, Xinxiang, Luoyang, Nanyang, Sanmenxia, Xuchang, Yuncheng and Zhumadian) were amplified by PCR. To obtain the complete S1 (1–789 aa) sequence, four primers (S1U1F, S1U1R, S1U2F and S1U2R) were designed and synthesised as previously described [28–30] according to Table 1, and the length of the final fragment was 2367 bp. The full-length ORF3 gene was obtained using previously published primers, and the length of the PCR product was 833 bp [31]. PCR products were purified, subcloned into pMD19-T and transformed using DH5α competent cells. The reagents were purchased from TaKaRa. Triplicate recombinant DNA positive clones from each PEDV isolate were sequenced by Sangon Biotech, China.

### Multiple sequence alignments and phylogenetic analysis

The complete S1 gene (2367 bp) was obtained through the sequence matching of S1U1 and S1U2 with flanking sequences removed. Sequences of S1 and ORF3 were processed by the Clustal W method through the DNAMAN 8 software, and phylogenetic trees were constructed by the neighbor-joining method using MEGA 6 software. Bootstrap values were indicated for each node from 1000 replicates. In addition, 37 reference strains (Table 2) were chosen from Genbank for inclusion in the phylogenetic analysis.

**Table 2:** Reference strains used in this study

Reference strains	Countries	S gene(nt)	ORF3 gene(nt)	Accession no.
CV777	Belgium,1978	2367	675	AF353511.1
CV777 attenuated	China, 1998	2364	276	KT323979.1
DR13/virulent	Korea,1999	2367	675	JQ023161.1
CH/ZMDZY/11	China,2011	2376	675	KC196276.1
LZC	China/Gansu,<2006	2367	675	EF185992.1
SM98	Korea,1998	2379	675	GU937797.1
MN	USA,2013	2376	675	KF468752.1
IA1	USA,2013	2376	675	KF468753.1
IA2	USA,2013	2376	675	KF468754.1
DR13/attenuated	Korea,2003	2364	276	JQ023162
SD-M	China/Shandong,2012	2364	276	JX560761
GD-A	China/Guangdong,2012	2376	675	JX112709
CH/S	China/Shanghai,1986	2367	675	JN547228
TC PC177-P2	USA,2013	1785	675	KM392229
BJ-2011-1	China/Beijing,2011	2376	675	JN825712
AH2012	China/Anhui,2012	2376	675	KC210145
AJ1102	China/Hubei,2011	2376	675	JX188454
83P-5	Japan1983	2364	—	AB548621
CH/JX-1/2013	China/Jiangxi,2013	2376	675	KF760557
CH/JX-2/2013	China/Jiangxi,2013	2376	675	KJ526096
Brl/87	France, 1987	2367	—	Z25483
HN1303	China/Luoyang,2013	2376	—	KR080551
CH/FCH-01	China,2013	—	675	KF476054(O)
CH/JCH	China,2013	—	675	KF476059(O)
CH/KF-01	China,2013	—	675	KF476051(O)
CH/XIP-03	China,2013	—	675	KF476058(O)
13-019349	USA,2013	2376	675	KF267450.1
Chinju99	Korea,2009	—	675	EU792474.1(O)
CH/HLJHH-2/2011	China,2012	—	675	JQ305099.1(O)
MK	Japan,2013	2367	675	AB548624.1(cS)
AD01	Korea,2013	2376	—	KC879280.1(cS)
CH/HNZZ/2011	China,2011	2364	—	JN601050(S1)
CH/FJXM-1/2012	China,2012	2376	—	JX070671
MYG-1/JPN/2014	Japan,2014	1794	—	LC063838.1
TTR-2/JPN/2014	Japan,2014	—	252	LC063828.1
OH851	USA,2014	2367	675	KJ399978.1
SH/2015/124	China,2015	2376	675	KU710245.1(S1), KU641672.1(ORF3)

## Results

### PEDV detection

PEDV were detected by PCR on 94.03% (63 of 67) pig farms in 17 cities, and 92.25% (119 of 129) of samples were positive for PEDV in clinical diseased samples.

### Phylogenetic analysis of the S1 gene

**Fig. 1:**
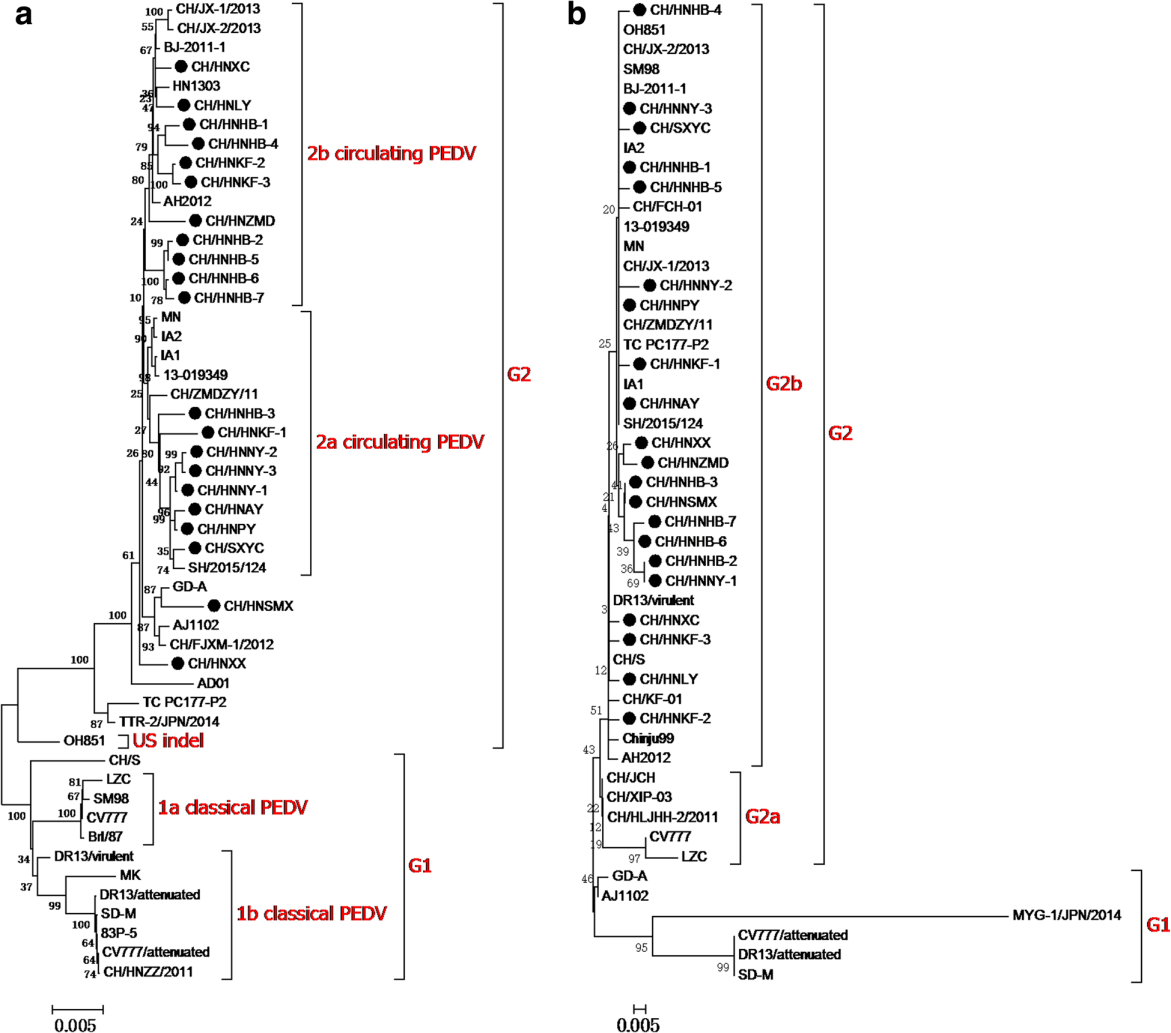
**a** Phylogenetic analysis of the S1 nucleotide sequences of 21 PEDV isolates, including the reference strains. **b** Phylogenetic analysis of the ORF3 nucleotide sequences of 21 PEDV isolates, including the reference strains. The trees were constructed by the neighbor-joining method in MEGA 6 software. Bootstrap values were indicated for each node from 1000 replicates. The names of the strains, years and places of isolation and GenBank accession numbers proposed are shown in Tables 2 and 3. ‘●’ indicates the strains in this study

According to the phylogenetic analysis of the S1 gene, 21 PEDV isolates in this study (Table 3) were all subtype G2 and were distributed in two subgroups: 2a circulating PEDV and 2b circulating PEDV (Fig. 1a). Our isolates showed a close relationship to some isolates from China (CH/ZMDZY/11, HN1303, AJ1102, et al.), Korea (AD01) and American (MN, IA1, IA2, 13–019349); however, our isolates differed from isolates collected previously from China (LZC, CH/HNZZ/2011, and SD-M), Korea (SM98), Japan (83-P5 and MK) and the vaccine strain CV777-attenuated (used in China) and DR13 (used in Korea) which showed similar to the phylogenetic trees based on the sequences of amino acid (see Additional file 1: Figure S1). According to the sequences of S1 genes processed by DNAMAN 8 software, our isolates exhibited 92.1–92.7% nucleotide identity and 89.7–91.2% amino acid identity compared with the CV777 strain. Meanwhile, our isolates exhibited 91.4–92.0% nucleotide identity and 89.1–90.7% amino acid identity compared with the CV777 attenuated strain.

**Table 3:** The PEDV field strains used in this study

Field strains	Origin	S gene(nt)	Accession no.	ORF3 gene(nt)	Accession no.
CH/HNHB-1	Hebi, Henan	2376	KU977480	675	KU977501
CH/HNHB-2	Hebi, Henan	2376	KU977481	675	KU977502
CH/HNHB-3	Hebi, Henan	2376	KU977482	675	KU977503
CH/HNHB-4	Hebi, Henan	2376	KU977483	675	KU977504
CH/HNHB-5	Hebi, Henan	2376	KU977484	675	KU977505
CH/HNHB-6	Hebi, Henan	2376	KU977485	675	KU977506
CH/HNHB-7	Hebi, Henan	2376	KU977486	675	KU977507
CH/HNKF-1	Kaifeng, Henan	2376	KU977487	675	KU977508
CH/HNKF-2	Kaifeng, Henan	2376	KU977488	675	KU977509
CH/HNKF-3	Kaifeng, Henan	2376	KU977489	675	KU977510
CH/HNSMX	Sanmenxia, Henan	2376	KU977490	675	KU977511
CH/SXYC	Yuncheng, Shanxi	2376	KU977491	675	KU977512
CH/HNXC	Xuchang, Henan	2376	KU977492	675	KU977513
CH/HNXX	Xinxiang, Henan	2376	KU977493	675	KU977514
CH/HNAY	Anyang, Henan	2376	KU977494	675	KU977515
CH/HNNY-1	Nanyang, Henan	2376	KU977495	675	KU977516
CH/HNNY-2	Nanyang, Henan	2376	KU977496	675	KU977517
CH/HNNY-3	Nanyang, Henan	2376	KU977497	675	KU977518
CH/HNZMD	Zhumadian, Henan	2376	KU977498	675	KU977519
CH/HNLY	Luoyang, Henan	2382	KU977499	675	KU977520
CH/HNPY	Puyang, Henan	2376	KU977500	675	KU977521

**Fig. 2:**
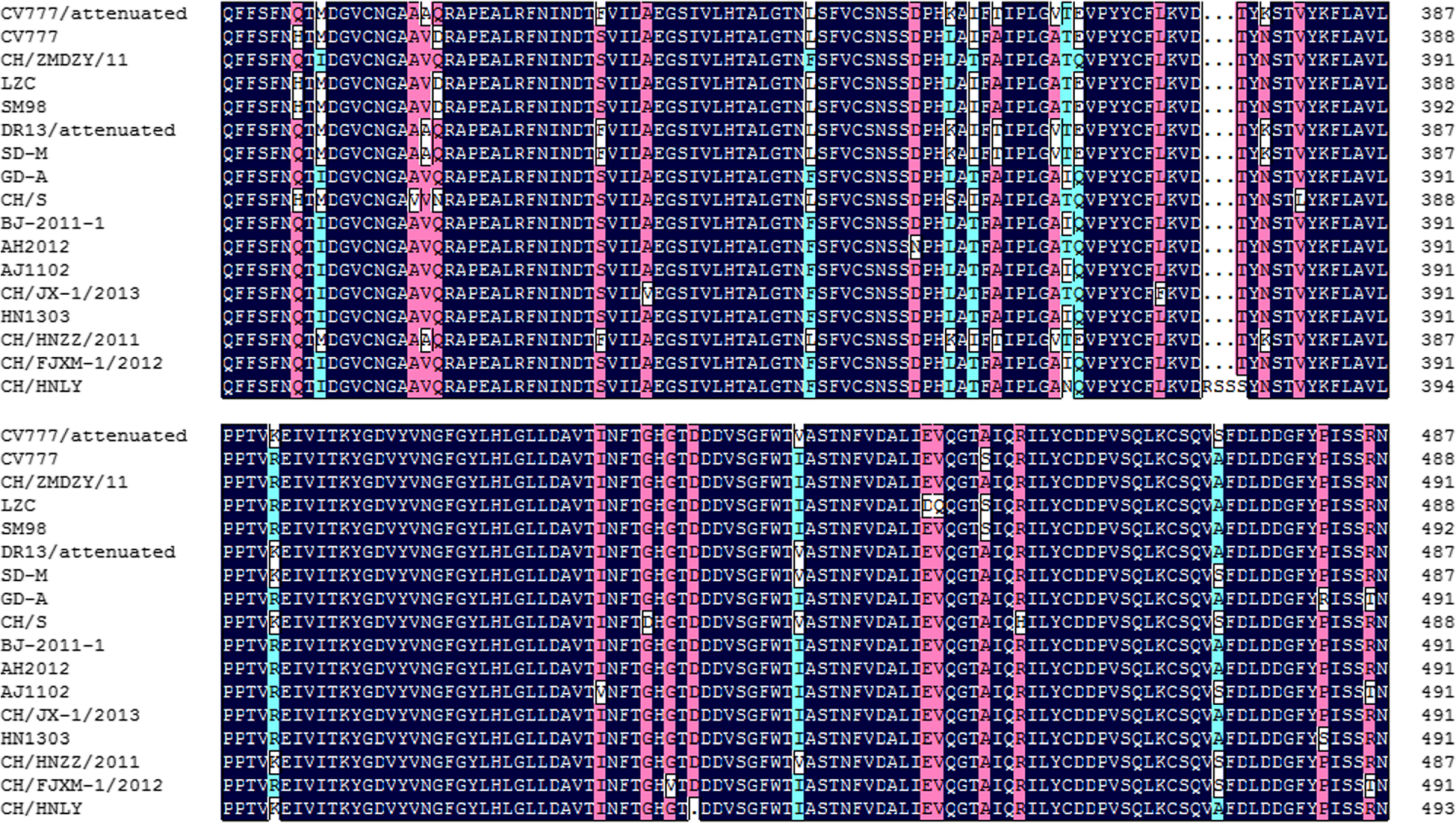
CV777/attenuated as the reference strain and the amino acid sequence of CH/HNLY, with 4-aa insertion/deletion (RSSS/T) at position 375 and 1-aa (D) deletion at position 430 (CV777 attenuated strain accession number: KT323979.1) are indicated

According to the predicted amino acid sequence of S1 genes in this study, we found one novel PEDV variant, CH/HNLY, that had 4-aa substitution at position 375, ^RSSS^375^T^ and a single deletion at position 430 (Fig. 2). Correspondingly, at the nucleotide level, CH/HNLY had 9-nt (GGTCGTCGT) insertion between positions 1123 (A) and 1124 (T) and 3-nt (GAT) deletion between positions 1283 (C) and 1287 (G).

We also examined the three major epitope regions, viz : aa 498–637, aa 747–754 and aa 763–770. The sequences at aa 747–754 were conserved between the latest Chinese PEDV isolates and CV777 attenuated strain, however the sequences at positions 498–637 and 763–770 were variable (Table 4).

**Table 4:** Analysis of amino acid mutations in epitopes domains of field strains and the CV777 attenuated vaccine strain (aa 498–637, aa 747–754 and aa 763–770 located in CV777 attenuated)

Strains	499	500	514	516	520	522	526	530	543	548	561	572	583	587	590	593	604	607	608	611	632	634	636	751	762	763	765	766	768
CV777 attenuated	T	L	S	A	H	G	I	T	R	T	S	K	K	S	L	G	E	S	G	F	Q	V	D	G	P	L	D	G	V
CV777					L	S	V										A			L	E	I				S	Y		
CH/ZMDZY/11				S	R		T			S			N			S					E					S	S		
CH/HNHB-1	D			S						S						S					E					S	S		
CH/HNHB-2, CH/HNHB-5			A	S						S						S	D		S		E					S	S		
CH/HNHB-3		P		S						S						S					E					S	S	D	
CH/HNHB-4	A			S						S		N				S					E					S	S		
CH/HNHB-6, CH/HNHB-7			A	S						S						S	D				E					S	S		
CH/HNKF-1, CH/HNLY				S						S						S					E					S	S		
CH/HNKF-2				S						S	Y					S					E					S	S		
CH/HNKF-3				S						S	Y				P	S					E					S	S		
CH/HNSMX										S				T		S					E		G			S	S		
CH/SXYC				S				A		S						S					E					S	S		
CH/HNXC				S						S						S					V					S	S		
CH/HNXX				S						S						S			V		E					S	S		
CH/HNAY	A			S	Y					S						S					E				L	S	S		
CH/HNNY-1, CH/HNNY-2, CH/HNNY-3,				S	Y					S						S					E					S	S		
CH/HNZMD				S						S		H				S			V		E					S	S		A
CH/HNPY	A			S	Y					S						S					E				L	S	S		

### Phylogenetic analysis of the ORF3 gene

According to the analysis of the ORF3 genes, 21 PEDV isolates in this study (Table 3) were all subtype G2 and were distributed in subgroup G2b which showed similar to the phylogenetic trees based on the sequences of amino acid (see Additional file 2: Figure S2); in addition, our strains showed a close relationship to published isolates and genetically differed from the vaccine strains which were all subtype G1 (Fig. 1b), indicating that the prevailing PEDV strains were mostly variants. According to the sequences of ORF3 genes processed by DNAMAN 8 software, our isolates exhibited 95.9–96.9% nucleotide identity and 93.8–96.4% amino acid identity compared with the CV777 strain. Meanwhile, our isolates exhibited 91.3–93.1% nucleotide identity and 84.6–89% amino acid identity compared with the CV777 attenuated strain.

## Discussion

Previous studies found that 79.66% of pig farms in 29 provinces to be positive for the presence of PEDV, with 72.27% of samples confirmed as PEDV-positive [16]. In the present study, 94.03% (63 of 67) of pig farms in 17 cities, 92.25% (119 of 129) samples, were PEDV-positive, indicating a high prevalence of PEDV in clinical diseased samples.

The S gene might correlate with PEDV pathogenicity [4]. The S1 domain of the S protein is the major target for PEDV vaccine development [32]. Our previous report suggested that the amino acid changes in the S1 domain might be associated with a change in antigenicity [20]. Extensive variation of the S protein has been reported earlier [17, 33]. In this study, we found that in the three major epitope regions, the amino acid sequence at aa 748–755 was conserved, whereas aa 499–638 and aa 764–771 were variable. Accordingly, within the S1 domain of the S protein, the series of single amino acid substitutions found were: ^S^516^A^, ^S^548^T^, ^S^593^G^, ^E^632^Q^, ^S^763^L^ and ^S^765^D^. These changes were found in the strain CH/ZMDZY/11 that was previously isolated in central China [34]. However, as shown in Table 4, there were other single aa mutations, suggesting that the gene encoding the antigenic domain of S1 may constantly vary. According to the phylogenetic analysis, our isolates were all subtype G2 (Fig. 1a) and mostly further divided into two subgroups, 2a circulating PEDV and 2b circulating PEDV. We have shown that the S1 domain of isolates differed genetically from the classical PEDV, but the isolates were similar to previously reported isolates from eastern, north western and southern China [35–37], which might be the reason why the currently existing vaccine is inefficient.

In this study, we detected one novel PEDV variant, CH/HNLY, with mutations at positions 375 and 430 (^RSSS^375^T^ and deletion at aa 430^D^), which are located on the receptor binding domain (aa 253–638) for pAPN [15, 38]. The phylogenetic analysis of nucleotide variation demonstrated that CH/HNLY was related to 2b circulating PEDV. It was reported that the classical PEDV exhibited weaker sugar-binding activity compared with the field isolate variant [39]. Reports have also found amino acid substitutions in the receptor-binding region [27]. Whether or not these changes affect the biological functions of PEDV will require further investigation.

The ORF3 gene is highly relevant to the virulence of PEDV [23, 40], since it regulates virus production [13]. The ORF3 genes of the PEDV isolates in this study and other isolates did not show the large deletion characteristic of the vaccine CV777 strain. According to the phylogenetic analysis of ORF3, the 21 PEDV isolates in this study were divided into subtype G2 (Fig. 1b). The ORF3 gene analysis not only suggested that the isolates in central China were not only vaccine-unrelated, but the presence of multiple, distinct mutations indicated there is widespread diversity in this virulence gene. However, further studies are needed to clarify whether the virulence is change among these PEDV strains.

Phylogenetic analysis of both the S1 and ORF3 genes showed that our isolates exhibited high similarity to variant reference strains and differed from CV777. Phylogenetic analysis of ORF3 did not reveal differences between our isolates and partial classical PEDV strains which was similar to other report [27]. Further studies are required to clarify the biological functions among PEDV phylogenetic groups.

## Conclusions

In conclusion, our study highlighted the present landscape of PEDV in central China, and the isolated strains in this study were all variable and genetically diverse. These findings make it clear that a new vaccine is required to control this disease. In addition, the discovery of a novel strain, CH/HNLY, provides an avenue for future investigations into the biological functions of PEDV.

## Additional files

Additional file 1: Figure S1. Phylogenetic analysis of the S1 amino acid sequences of 21 PEDV isolates, including the reference strains. The trees were constructed by the neighbour-joining method in MEGA 6. Bootstrap values were indicated for each node from 1000 replicates. The names of the strains, years and places of isolation and GenBank accession numbers proposed are shown in Tables 2 and 3. ‘●’ indicates the strains in this study. The phylogenetic analysis of CH/HNLY (with 4-aa insertion/deletion (RSSS/T) at position 375 and 1-aa (D) deletion at position 430) was showed. (TIF 2238 kb)
Additional file 2: Figure S2. Phylogenetic analysis of the ORF3 amino acid sequences of 21 PEDV isolates, including the reference strains. The trees were constructed by the neighbour-joining method in MEGA 6. Bootstrap values were indicated for each node from 1000 replicates. The names of the strains, years and places of isolation and GenBank accession numbers proposed are shown in Tables 2 and 3. ‘●’ indicates the strains in this study. (TIF 1880 kb)
