# Supplementary figures and images for: Detection and phylogenetic analysis of porcine epidemic diarrhea virus in central China based on the ORF3 gene and the S1 gene
Source: Virol J. 2016 Nov 25;13:192. doi: 10.1186/s12985-016-0646-8 (PMC5123408; doi:10.1186/s12985-016-0646-8)

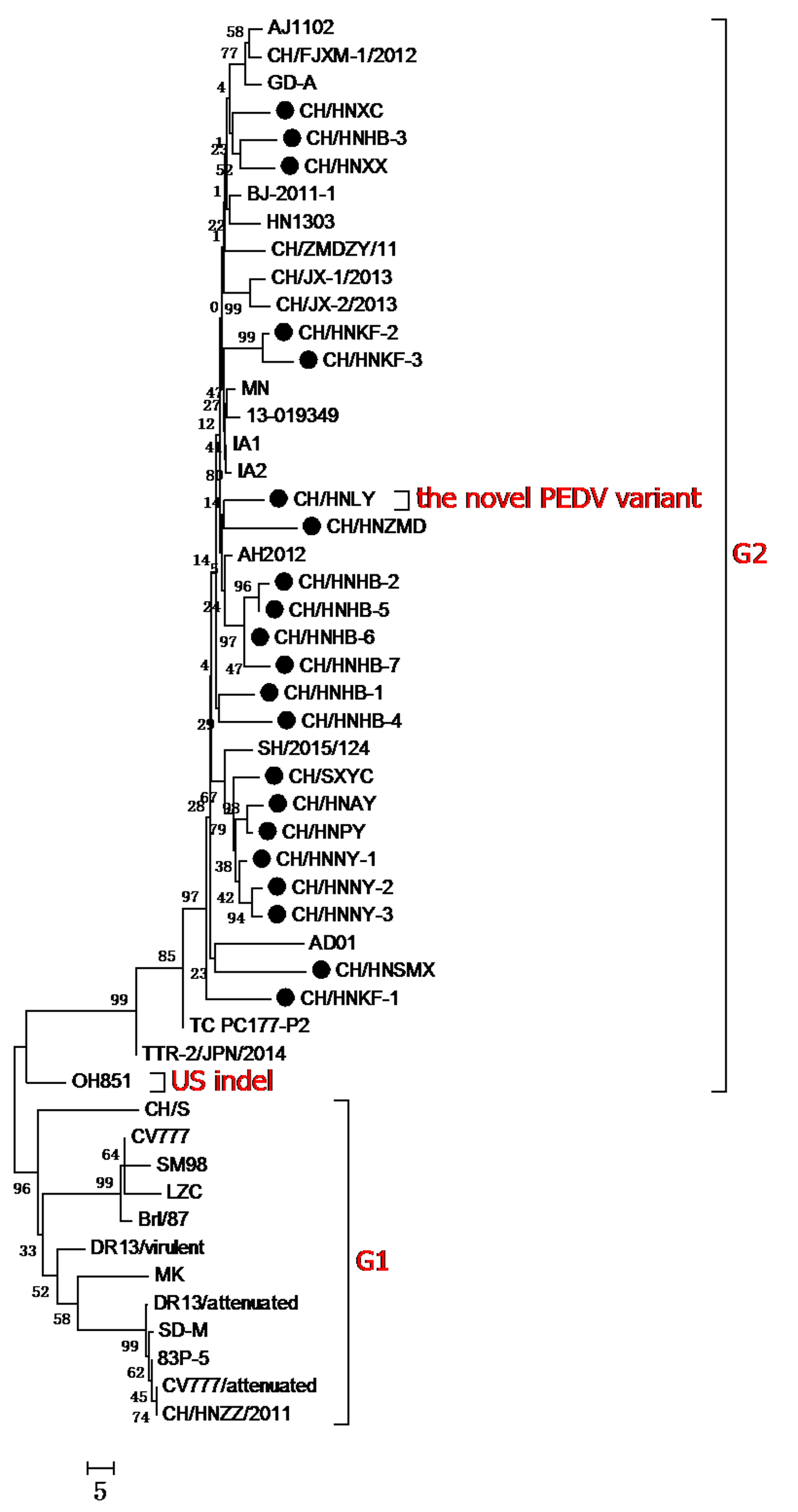

Supplement: Additional file 1: Figure S1. — Phylogenetic analysis of the S1 amino acid sequences of 21 PEDV isolates, including the reference strains. The trees were constructed by the neighbour-joining method in MEGA 6. Bootstrap values were indicated for each node from 1000 replicates. The names of the strains, years and places of isolation and GenBank accession numbers proposed are shown in Tables 2 and 3. ‘●’ indicates the strains in this study. The phylogenetic analysis of CH/HNLY (with 4-aa insertion/deletion (RSSS/T) at position 375 and 1-aa (D) deletion at position 430) was showed. (TIF 2238 kb) [file 12985_2016_646_MOESM1_ESM.tif]

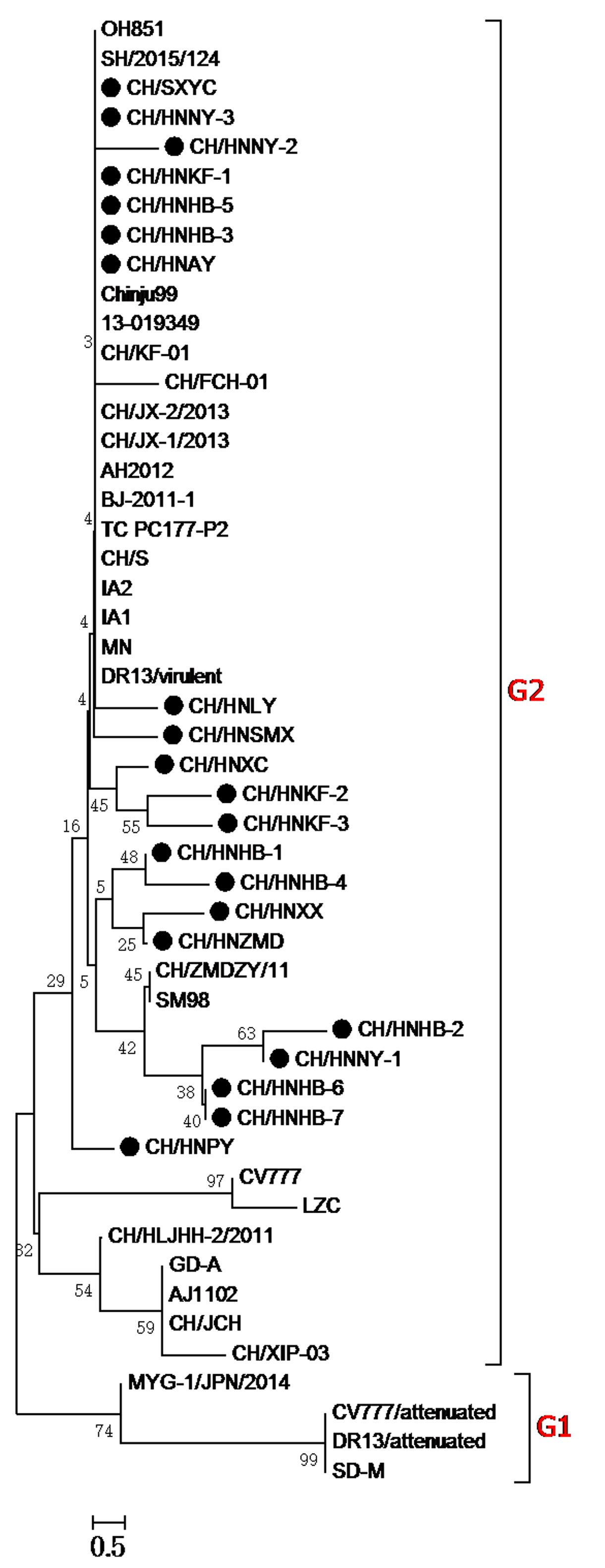

Supplement: Additional file 2: Figure S2. — Phylogenetic analysis of the ORF3 amino acid sequences of 21 PEDV isolates, including the reference strains. The trees were constructed by the neighbour-joining method in MEGA 6. Bootstrap values were indicated for each node from 1000 replicates. The names of the strains, years and places of isolation and GenBank accession numbers proposed are shown in Tables 2 and 3. ‘●’ indicates the strains in this study. (TIF 1880 kb) [file 12985_2016_646_MOESM2_ESM.tif]
